# Elevated protein levels and mRNA upregulation of Lp**-**PLA_2_, IL-6, and TNF-α in cardiovascular disease among a Saudi population

**DOI:** 10.3389/fcvm.2026.1774428

**Published:** 2026-05-08

**Authors:** Ziad H. Al-Oanzi, Fawaz O. Alenazy, Zien A. Alhashash, Raghad A. Alshammari, Fouz M. Alruwaili, Aryaf H. Alruwaili, Aryam S. Alruwaili, Ftoon N. Al-Shammri, Shumukh H. Alfuhaigi, Hala M. Alrwily, Mansour M. Alharbi, Syed M. Shahrukh, Taha H. Noor, Najeebullah Bugti

**Affiliations:** 1 Department of Clinical Laboratory Sciences, Colleges of Applied Medical Sciences, Jouf University, Sakaka, Saudi Arabia; 2 Cardiac Center, King Abdul Aziz Specialist Hospital, Sakaka, Saudi Arabia; 3 Ministry of Health, Aljouf Health Cluster, Sakaka, Saudi Arabia

**Keywords:** atherosclerosis, cytokines, gene expression, inflammation, Lp-PLA_2_, oxidized LDL, Saudi population

## Abstract

**Background:**

Lp-PLA_2_ connects oxidised lipid metabolism to vascular inflammation, however data from Middle Eastern populations remain limited. This study evaluated circulating Lp-PLA_2_, inflammatory cytokines, oxidized lipids, and whole-blood mRNA expression in a Saudi cohort.

**Methods:**

A case–control study was conducted including 30 males with established atherosclerotic cardiovascular disease (CVD) and 30 males without known CVD. Plasma biomarkers were measured using ELISA, and whole-blood mRNA expression of Lp-PLA_2_, IL-6, and TNF-α was assessed using RT-qPCR. Multivariable linear regression analyses were performed adjusting for BMI, LDL-C, HbA1c, and smoking.

**Results:**

Compared with non-CVD participants, individuals with CVD showed higher Lp-PLA_2_ (419 ± 188 vs. 101 ± 32 ng/mL; *P* < 0.001), IL-6 (74 ± 17 vs. 40 ± 19 pg/mL; *P* < 0.001), TNF-α (4.4 ± 0.9 vs. 3.7 ± 0.8 pg/mL; *P* < 0.01), ox-LDL (430 ± 143 vs. 242 ± 67 ng/mL; *P* < 0.001), and LP-a (134 ± 31 vs. 90 ± 33 nmol/L; *P* < 0.001), with a more atherogenic lipid profile (LDL 3.02 ± 0.63 vs. 1.72 ± 0.63 mmol/L; *P* < 0.001; HDL lower: *P* < 0.01). mRNA expression of Lp-PLA_2_, IL-6, and TNF-α was upregulated in CVD. Lp-PLA_2_ correlated with IL-6 (*r* = 0.75, *P* < 0.001), TNF-α (*r* = 0.36, *P* < 0.01), ox-LDL (*r* = 0.85, *P* < 0.001), and LDL (*r* = 0.80, *P* < 0.001). However, in multivariable regression analyses, LDL-C and HbA1c were significant predictors of biomarker levels. Although LDL-C and HbA1c were associated with biomarker levels after adjustment, residual confounding related to disease status and treatment exposure cannot be excluded.

**Conclusions:**

Elevated inflammatory and oxidative biomarkers in CVD are strongly associated with underlying metabolic factors, particularly dyslipidemia and glycemic status. These findings support Lp-PLA_2_ as a marker of lipid-associated inflammatory activity rather than an independent causal mediator. This study provides population-specific data from a Saudi cohort and highlights the importance of metabolic context in interpreting cardiovascular biomarkers.

## Introduction

A complex interaction between dysregulated lipid metabolism, persistent vascular inflammation, and maladaptive tissue remodeling drives cardiovascular disease (CVD), which remains the leading cause of morbidity and death globally (1). Atherosclerosis, the pathological basis of most CVD, is increasingly recognized as an inflammatory disorder in which oxidative modification of lipoproteins and immune activation act in concert to promote plaque development and instability (2–4).

**Figure 1:**
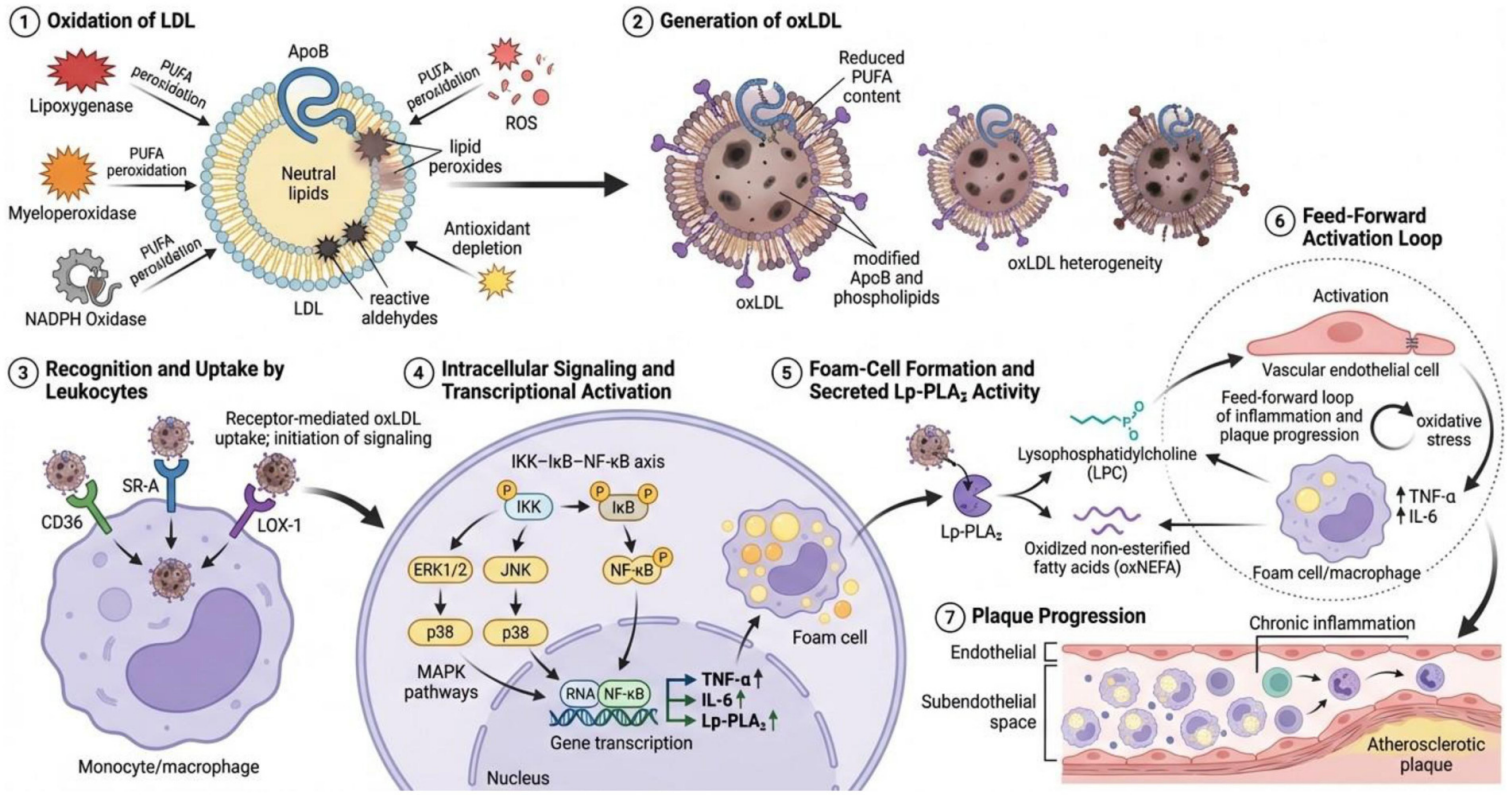
Schematic overview of oxidized LDL–mediated inflammatory signaling and Lp-PLA_2_–associated lipid remodeling in atherosclerosis.

An important molecule in the relationship between inflammation and lipid oxidation is lipoprotein-associated phospholipase A_2_ (Lp-PLA_2_), which is also called platelet-activating factor acetylhydrolase (PAF-AH) or group VIIA phospholipase A_2_ and is encoded by the PLA2G7 gene (5). Lp-PLA_2_ is a secreted, calcium-independent serine hydrolase enzyme produced primarily by macrophages and other inflammatory cells. It is found in the bloodstream bound mainly to low-density lipoprotein (LDL) molecules, with a smaller proportion bound to high-density lipoprotein (HDL) (5–7). Oxidative alteration of LDL produces oxidized LDL (ox-LDL), a significant factor in vascular inflammation and the formation of atherosclerotic plaques. Lipoprotein endogenous antioxidants are depleted, both those acted upon by enzymatic oxidation systems—including lipoxygenase, myeloperoxidase, and NADPH oxidase—and those acted upon by non-enzymatic auto-oxidation processes, promoting the peroxidation of polyunsaturated fatty acids (PUFAs) (8–10). These reactions produce lipid peroxides and reactive aldehydes that modify apolipoprotein B and the phospholipid components of LDL, producing heterogeneous ox-LDL molecules with varying lipid content and different receptor-binding properties (8, 10). Ox-LDL is preferentially recognized by leukocyte scavenging receptors, such as Cluster of Differentiation 36 (CD36), scavenging receptor-A (SR-A), and oxidized lectin-like LDL receptor-1 (LOX-1), facilitating its cellular uptake and stimulating intracellular inflammatory signaling (10, 11). The binding of oxidized low-density lipoprotein (ox-LDL) molecules to these receptors activates key inflammatory pathways, including several axons such as IKK–IκB–NF-κB and the stress-responsive mitogen-activated protein kinase (MAPK) pathway (ERK1/2, JNK, and p38) (12–14). This activation, in turn, stimulates several pathways, including the transcription of inflammatory mediators such as tumor necrosis factor-alpha (TNF-α), interleukin-6 (IL-6), and Lp-PLA_2_. This, in turn, leads to lipid buildup in macrophages and the development of foam cells (8, 15, 16). Lysophosphatidylcholine (LPC) and oxidized non-esterified fatty acids (oxNEFAs) are byproducts of the breakdown of oxidized phospholipids on ox-LDL by Lp-PLA_2_ (5, 6, 17–19). These lipid mediators exert pro-inflammatory effects on endothelial cells, promote cytokine release from macrophages, and amplify oxidative stress, collectively forming an integrated inflammatory network that links the burden of oxidized lipids to persistent vascular inflammation and plaque development (11, 20–23) (Figure 1).

According to the findings of major observational studies and meta-analyses, there is a important association between an increase in the circulating Lp-PLA_2_ mass or activity and the development of subclinical atherosclerosis, the presence of unfavorable plaque features, and an elevated risk of CHD (coronary heart disease) and ischemic stroke. This implication holds true even when controlling traditional cardiovascular risk factors (24–26). However, Lp-PLA_2_ mass and enzymatic activity capture partially distinct biological information and may diverge according to lipoprotein distribution, substrate availability, and inflammatory state (26, 27). Histopathological and imaging studies further demonstrate that Lp-PLA_2_ is enriched within macrophage-rich, lipid-laden regions of atherosclerotic plaques and co-localizes with oxidatively modified LDL, supporting an association with necrotic core formation and plaque vulnerability (28).

The function of Lp-PLA_2_ in atherosclerosis remains incompletely elucidated, despite the existence of these relationships. In substantial phase III clinical studies, including STABILITY and SOLID-TIMI 52, darapladib’s pharmacological inhibition of Lp-PLA_2_ did not lead to a significant reduction in severe adverse cardiovascular events, while effectively suppressing enzyme activity (29, 30). Simultaneously, genetic investigations focusing on loss-of-function variants of PLA2G7, including the V279F null allele common in East Asian populations, have produced inconsistent or negligible correlations with coronary risk (31, 32). The results suggest that Lp-PLA_2_ may primarily serve as a marker of disease activity or function in a context-dependent manner, rather than functioning as a universal causal factor of atherosclerosis. Recent studies have further highlighted the interplay between lipid metabolism, inflammatory signaling, and cardiovascular risk across diverse populations (33–36).

Experimental studies nevertheless provide biologically plausible mechanisms through which Lp-PLA_2_-derived lipid mediators may contribute to vascular inflammation. LPC and selected oxNEFAs have been shown to promote endothelial activation, upregulate adhesion molecules and inflammatory cytokines, impair endothelial barrier function and vasorelaxation, and modulate macrophage and vascular smooth muscle cell phenotypes (8, 9, 37). Importantly, the biological effects of Lp-PLA_2_ appear to depend on its lipoprotein carrier. LDL-associated Lp-PLA_2_ may preferentially generate pro-inflammatory lipid mediators within the arterial wall, whereas HDL-associated Lp-PLA_2_ has been proposed, under specific conditions, to participate in detoxification of oxidized phospholipids. The complex functional role of LP-a lipoprotein within the vascular environment is further complicated by the abundance of oxidized phospholipids, which may influence the inflammatory consequences that follow Lp-PLA_2_ activity (6, 38, 39).

Based on these findings, many important questions need answering, such as how substrate availability and the type of carrier lipoprotein affect Lp-PLA_2_ activity in living organisms, how this enzyme's lipid mediators regulate inflammatory signaling in immune cells that circulate in the blood, and when Lp-PLA_2_ is only a disease burden marker and not really involved in the pathophysiology of atherosclerosis. Particularly for communities that are still underrepresented in cardiovascular biomarker studies, resolving these disparities is crucial.

Therefore, this study aimed to compare circulating Lp-PLA_2_ protein levels, whole blood gene expression, and inflammatory cytokines and lipid peroxidation markers in patients with atherosclerotic CVD to non-CVD participants. This research uses biochemical, inflammatory, and molecular techniques to evaluate Saudi lipid oxidation and inflammation pathways related to cardiovascular disease.

## Methods

### Design and participants

This comparative case–control study was managed at King Abdulaziz Specialist Hospital, Sakaka, Saudi Arabia. The study population comprised 30 males with atherosclerotic CVD (mean age 55 ± 8 years) and 30 males non-CVD participants (mean age 53 ± 14 years). This research deliberately selected exclusively male participants. In Saudi society, the prevalence of smoking is significantly greater among males than females; thus, restricting recruitment to males facilitated a more precise characterization of smoking-related metabolic and inflammatory risk factors linked to atherosclerotic cardiovascular disease, while reducing sex-related behavioral variability. Sex-specific differences in lipid metabolism and inflammatory responses are well established in cardiovascular disease. The restriction to male participants was intended to reduce heterogeneity in this exploratory cohort; however, the approach limits generalizability, and future studies should include both sexes. Based on diagnostic and clinical records, many patients with atherosclerotic cardiovascular disease presented with comorbid metabolic and cardiovascular conditions, such as diabetes, dyslipidemia, hypertension, and overweight or obesity, and the majority were former smokers. In comparison, participants without established CVD, although some had cardiometabolic risk factors including smoking, hypertension, and diabetes. Patients with atherosclerotic cardiovascular disease were handled in accordance with conventional clinical practice, as shown by the diagnostic records and treatment regimens. Many patients were given metformin for glycemic regulation, in conjunction with drugs aimed at lowering blood pressure and cholesterol levels. Plasma samples were collected from CVD patients at the Cardiology Center and from healthy volunteers at the Care Center. Samples obtained from participants who died during the study period prior to completion of laboratory analyses. Project No. (2023 127) received Institutional Review Board (IRB) clearance after it was approved by the Research Ethics Committee of the Ministry of Health, Al Qurayyat Health Affairs, and the National Committee for Bioethics and Medicine (H 13 S 071).

### Blood collection, processing, and storage

Blood samples were collected from the veins and plasma was centrifuged according to routine laboratory techniques after the patient and health control fasted overnight. Separate samples of the plasma were prepared for regular haematological and biochemical analyses to be performed immediately and for storage at −80 °C to be used for the subsequent examination of cardiovascular biomarkers. To prevent interference with biomarker measurements, samples that exhibited visible hemolysis (pink to red plasma discoloration) were excluded and screened for hemolysis through visual inspection.

### Biochemical and hematological analyses

The clinical laboratories of the hospital assessed the lipid profile, including total cholesterol (TC), triglycerides (TG), LDL-C, and HDL-C utilizing enzymatic method spectrophotometric assays. Measurements of alanine aminotransferase (ALT), creatinine, and urea were conducted to offer further insights into renal and liver function, respectively. The parameters of the comprehensive blood count (CBC) and haemoglobin levels were also measured.

### Cardiovascular, inflammatory, and oxidative biomarkers

For the purpose of analyzing several parameters, including TNF-α, IL-6, FABP3, ox-LDL, Lp-PLA_2_ and LP-a, this second plasma sample was kept at −80 °C until these parameters were analyzed. The ELISA kits (Elabscience®, Houston, Texas, USA) were used to test all biomarkers according to the methods provided by the manufacturers. Intra- and inter-assay coefficients of variation were <5%, supporting high analytical precision.

### Lp-PLA_2_ (PLA2G7) mRNA expression by RT-qPCR

For gene-expression analyses, whole blood was collected in EDTA tubes. Total RNA was extracted using an RNA purification kit (Genaxxon bioscience, Germany). RNA concentration and purity were assessed by spectrophotometry (A260/A280 ratio); values within an acceptable range were required to proceed. The Scriptase RT cDNA kit from Genexxon Bioscience in Germany was used to generate first strand cDNA in a 20 µL reaction using RNase free water. To inactivate reverse transcriptase, reverse transcription was carried out at 50 °C for 10 min, followed by 95 °C for 10 min. The RT-PCR was performed using an AriaMx instrument (Agilent Technologies, Santa Clara, CA, USA) and the following primers were designed and are listed in the Supplementary Material. All reactions were performed in duplicate using PCR master mix (Genaxxon Bioscience). GAPDH served as the reference gene for normalization and was included as a calibrator as specified. Relative expression was calculated by the 2^−ΔΔCt^ method.

### Statistical analysis

Continuous variables are presented as mean ± standard deviation (SD). Normality was assessed using visual inspection and appropriate statistical tests. Group comparisons were performed using independent *t*-tests for normally distributed variables and non-parametric tests where appropriate. Correlations were assessed using Pearson or Spearman methods as applicable. To account for potential confounding, multivariable linear regression analyses were performed with Lp-PLA_2_, IL-6, and TNF-α as dependent variables. Covariates were selected *a priori* based on biological relevance and included body mass index (BMI), LDL-C, HbA1c, and smoking status. Given the modest sample size (*n* = 60), parsimonious models were used to minimize overfitting. Regression results are presented as *β* coefficients with 95% confidence intervals (CI). A two-sided *P*-value <0.05 was considered statistically significant.

## Results

### Fundamental demographic and clinical characteristics

Table 1 summarizes the standard demographic and clinical parameters. While there was no difference in age between the groups, there was a significant difference in body mass index (BMI) between non-CVD participants and CVD patients (29 ± 5 vs. 26 ± 1 kg/m^2^; *P* = 0.01). Smoking exposure was more frequent in the CVD group. Cardiometabolic comorbidities—including hypertension, diabetes mellitus, and dyslipidemia—were highly prevalent among CVD patients, while less prevalent in non-CVD participants. Established cardiovascular conditions and cardiovascular medications were confined to the CVD cohort.

**Table 1:** Demographic characteristics, cardiovascular comorbidities, and medication Use in patients with CVD and the participants without established CVD.

Variable	CVD (*n* = 30)	Non-CVD (*n* = 30)	*P* value
Demographic characteristics			
Age (years)	55 ± 1	53 ± 1	—
Body mass index (kg/m^2^)	29 ± 5	26 ± 1	0.01
Lifestyle factors			
Smoking status, *n* (%)			
Current smoker	12 (40.0%)	7 (23.3%)	—
Former smoker	16 (53.3%)	11 (36.7%)	—
Never smoker	2 (6.7%)	12 (40.0%)	—
Metabolic and cardiovascular comorbidities			
Hypertension, *n* (%)	28 (93.3%)	9 (30.0%)	—
Diabetes mellitus, *n* (%)	30 (100.0%)	7 (23.3%)	—
Dyslipidemia, *n* (%)	30 (100.0%)	4 (13.3%)	—
Established cardiovascular conditions (patients only)			
Ischemic heart disease (IHD)	14 (46.7%)	—	NA
Stroke	2 (6.7%)	—	NA
Atrial fibrillation (AF)	2 (6.7%)	—	NA
Chronic kidney disease (CKD)	5 (16.7%)	—	NA
Medications (patients only)			
Aspirin	26 (86.7%)	—	NA
Clopidogrel	3 (10.0%)	—	NA
Insulin intake	30 (100%)	—	NA
Statins	30 (100.0%)	—	NA
Beta-blockers	28 (93.3%)	—	NA
ACE inhibitors (ACEI)	29 (96.7%)	—	NA
Apixaban	4 (13.3%)	—	NA

### Baseline: clinical, biochemical, and hematological characteristics

As shown in Table 2, CVD patients exhibited a significantly more adverse metabolic profile. Fasting glucose was higher in CVD patients than in non-CVD participants (10.8 ± 3.5 vs. 5.8 ± 1.5 mmol/L; *P* = 0.001). The lipid profile was distinctly atherogenic—TC 4.2 ± 0.6 vs. 3.6 ± 0.5 mmol/L (*P* < 0.001), LDL C 3.0 ± 0.6 vs. 1.8 ± 0.6 mmol/L (*P* < 0.0001), and lower HDL 1.1 ± 0.1 vs. 1.3 ± 0.3 mmol/L (*P* < 0.001); TG were similar between groups. Liver and kidney indices were modestly higher in CVD (ALT 38 ± 30 vs. 22 ± 7 IU/L, *P* < 0.05; creatinine 107 ± 24 vs. 81 ± 12 μmol/L, *P* < 0.001; urea 10 ± 5 vs. 4.8 ± 1.2 mmol/L, *P* < 0.01). Hematological characteristics: CVD exhibited a pro-inflammatory blood profile with higher WBC (10.6 ± 5.0 vs. 7.0 ± 1.6 × 10^3^/μL; *P* < 0.001), neutrophilia (65 ± 11% vs. 47 ± 11%; *P* < 0.0001) and relative lymphopenia (22 ± 11% vs. 40 ± 10%; *P* < 0.0001). Red cell indices differed (MCV, MCH higher; MCHC lower; all *P* ≤ 0.01), and MPV was reduced (8.0 ± 1.2 vs. 10 ± 0.6 fL; *P* < 0.001).

**Table 2:** Clinical, biochemical, and hematological characteristics of the study population.

Variable	Non-CVD (*n* = 30)	CVD (*n* = 30)	*P* value
Biochemical/lipid profile			
Glucose (mmol/L)	5.8 ± 1.5	10.8 ± 3.5	0.001
Total cholesterol (mmol/L)	3.6 ± 0.5	4.2 ± 0.6	0.001
HDL-C (mmol/L)	1.3 ± 0.3	1.1 ± 0.1	0.001
LDL-C (mmol/L)	1.8 ± 0.6	3.0 ± 0.6	<0.001
Triglycerides (mmol/L)	1.3 ± 0.5	1.3 ± 0.5	NS
ALT (IU/L)	22 ± 7	38 ± 30	0.05
Creatinine (µmol/L)	81 ± 12	107 ± 24	0.001
Urea (mmol/L)	4.8 ± 1.2	10.0 ± 5.0	0.01
Hematological parameters			
WBC (×10^3^/µL)	7.0 ± 1.6	10.6 ± 5.0	0.001
RBC (×10⁶/µL)	5.0 ± 0.35	4.7 ± 0.9	—
Hematocrit (%)	43 ± 3	43 ± 8	—
MCV (fL)	83 ± 4	90 ± 3.7	<0.001
MCH (pg)	27 ± 2	29 ± 2	0.01
MCHC (g/dL)	33 ± 1.2	32 ± 1.3	0.01
RDW-CV (%)	13 ± 1.5	12 ± 2.0	0.05
MPV (fL)	10.0 ± 0.6	8.0 ± 1.2	0.001
Platelets (×10^3^/µL)	268 ± 50	275 ± 103	—
Neutrophils (%)	47 ± 11	65 ± 11	<0.001
Lymphocytes (%)	40 ± 10	22 ± 11	<0.001
Monocytes (%)	8.0 ± 1.8	8.6 ± 2.3	—
Eosinophils (%)	3.0 ± 1.3	2.3 ± 1.3	0.05
Basophils (%)	0.7 ± 0.2	0.8 ± 0.3	0.05

### Inflammatory and oxidative biomarkers

In Table 3, across the full biomarker panel, CVD patients had higher IL-6 (74 ± 17 vs. 40 ± 17 pg/mL; *P* < 0.0001), higher TNF-α (4.3 ± 0.9 vs. 3.6 ± 0.8 pg/mL; *P* < 0.0001), higher ox-LDL (430 ± 147 vs. 242 ± 66 ng/mL; *P* < 0.0001), higher LP-a (134 ± 31 vs. 90 ± 33 nmol/L; *P* < 0.0001), and markedly higher Lp-PLA_2_ (419 ± 185 vs. 101 ± 31 ng/mL; *P* < 0.0001). FABP3 trended higher without statistical significance (3.4 ± 1.0 vs. 3.1 ± 0.1 ng/mL; *P* = 0.073).

**Table 3:** Estimated levels of cardiovascular disease markers among patients and the participants without established CVD.

Measure	Non-CVD (mean ± SD)	CVD (mean ± SD)	*P*-value
IL-6 (pg/mL)	40 ± 17	74 ± 17	0.0001
TNF-α (pg/mL)	3.6 ± 0.8	4.3 ± 0.9	0.0001
ox-LDL (ng/mL)	242 ± 66	430 ± 147	0.0001
FABP3 (ng/mL)	3.1 ± 0.1	3.4 ± 1	0.072
LP-a (nmol/L)	90 ± 33	134 ± 31	0.0001
Lp-PLA2 (ng/mL)	101 ± 31	419 ± 185	0.0001

### Multivariable regression analysis

In Table 4, multivariable linear regression models adjusted for BMI, LDL-C, HbA1c, and smoking showed that LDL-C and HbA1c were consistent significant predictors of Lp-PLA_2_ and inflammatory biomarkers. However, residual confounding related to disease status and treatment exposure cannot be excluded. LDL-C was strongly associated with Lp-PLA_2_ and IL-6, while HbA1c independently predicted both Lp-PLA_2_ and IL-6 levels. Smoking was associated with IL-6 and TNF-α. After adjustment, the categorical CVD status was not retained as an independent predictor in the final models.

**Table 4:** Multivariable linear regression for (A) Lp-PLA_2_ (ng/mL), (B) IL-6 (pg/mL) and (C) TNF-α (pg/mL).

Variable	*β* Coefficient	95% CI	*P*-value
(A)			
CVD (1 vs. 0)	Not retained	—	—
BMI (kg/m^2^)	5.62	1.22 to 10.02	0.013
LDL-C (mmol/L)	67.23	47.92 to 86.53	<0.001
HbA1c (%)	17.98	6.18–29.77	0.004
Smoking (yes)	56.65	−8.38 to 121.67	0.086
(B)			
CVD (1 vs. 0)	Not retained	—	—
BMI (kg/m^2^)	0.54	−0.25 to 1.32	0.174
LDL-C (mmol/L)	3.87	0.44 to 7.31	0.028
HbA1c (%)	2.38	0.28 to 4.48	0.027
Smoking (yes)	12.01	0.44 to 23.58	0.042
(C)			
CVD (1 vs. 0)	Not retained	—	—
BMI	−0.017	−0.049 to 0.016	0.315
LDL-C	0.13	−0.02 to 0.27	0.083
HbA1c	0.08	−0.01 to 0.16	0.085
Smoking	0.51	0.02–0.99	0.041

Multivariable linear regression models were adjusted for BMI, LDL-C, HbA1c, and smoking. Due to collinearity between CVD status and metabolic variables, the CVD variable was not retained as an independent predictor in the final models.

### Correlation between protein-mRNA analyses

In Table 5, concordant differences at both protein and transcript levels were observed for Lp-PLA_2_: protein 419 ± 185 vs. 101 ± 31 ng/mL (*P* < 0.0001); mRNA 1.243 ± 0.102 vs. 1.021 ± 0.130 (*P* < 0.0001). Within CVD, protein–mRNA correlation was significant (*r* = 0.627; *P* < 0.001); not in non-CVD participants (*r* = 0.19; *P* < 0.2). IL-6: protein 74 ± 17 vs. 40 ± 17 pg/mL (*P* < 0.0001); mRNA 1.920 ± 0.340 vs. 1.213 ± 0.381 (*P* < 0.0001). Strong protein–mRNA coupling in CVD (*r* = 0.778; *P* < 0.0001), not in non-CVD participants (*r* = 0.24; *P* = 0.1). TNF-α: protein 4.3 ± 0.9 vs. 3.6 ± 0.8 pg/mL (*P* < 0.01); mRNA 1.25 ± 0.10 vs. 1.05 ± 0.15 (*P* < 0.0001). Protein–mRNA correlation in CVD was significant (*r* = 0.612; *P* < 0.001); not in non-CVD participants (*r* = 0.17; *P* < 0.2). Overall, these findings indicate that coupling between transcriptional activity and circulating protein levels for Lp-PLA_2_, IL-6, and TNF-α is disease dependent, being present in CVD but less prevalent in non-CVD participants.

**Table 5:** Protein levels, mRNA expression, and protein–mRNA correlations of Lp-PLA_2_, IL-6, and TNF-α.

Parameter	Lp-PLA_2_	IL-6	TNF-α
Protein level (CVD)	419 ± 185 ng/mL	74 ± 17 pg/mL	4.3 ± 0.9 pg/mL
Protein level (Non-CVD)	101 ± 31 ng/mL	40 ± 17 pg/mL	3.6 ± 0.8 pg/mL
Protein *P* value	<0.0001	<0.0001	<0.01
mRNA expression (CVD)	1.243 ± 0.102	1.920 ± 0.340	1.25 ± 0.10
mRNA expression (Non-CVD)	1.021 ± 0.130	1.213 ± 0.381	1.05 ± 0.15
mRNA *P* value	<0.0001	<0.0001	<0.0001
Protein–mRNA correlation (CVD)	*r* = 0.627	*r* = 0.778	*r* = 0.612
	*P* < 0.001	*P* < 0.0001	*P* < 0.001
Protein–mRNA correlation (Non-CVD)	*r* = 0.19	*r* = 0.24	*r* = 0.17
	*P* = 0.29	*P* = 0.18	*P* = 0.28

Values are expressed as mean ± SD. Protein concentrations were measured by ELISA, and mRNA expression was quantified by RT-qPCR using the 2^−ΔΔCt^ method normalized to GAPDH. Correlations were assessed using Pearson's correlation coefficient.

### Associations between Lp-PLA_2_ concentration and circulating lipid profiles, inflammatory, and oxidative biomarkers

**Figure 2:**
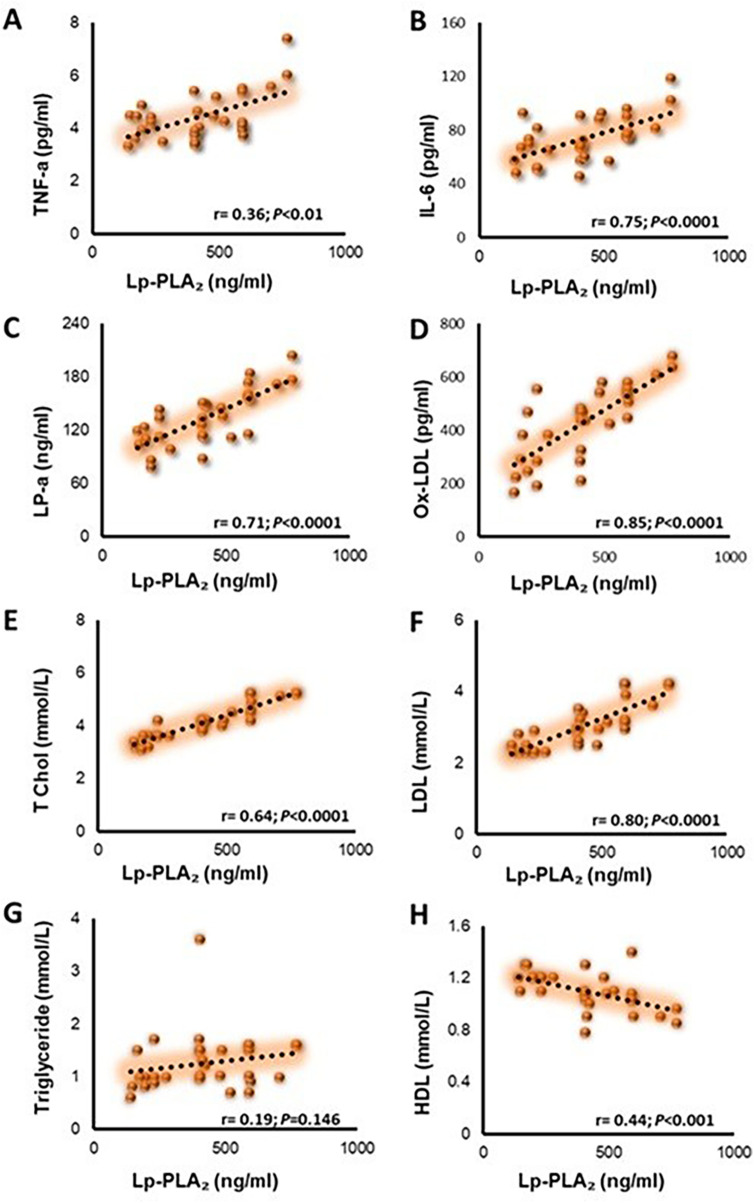
Correlation of Lp-PLA_2_ with inflammatory and lipid markers in pooled biochemical data. Panels show scatter plots of Lp-PLA2 (*x*-axis) vs. each marker (*y*-axis) with a least-squares regression line. Numbers on each panel are Pearson's *r* and two-sided *P*-values. **(A)** TNF-α: *r* = 0.36; *P* < 0.01, **(B)** IL-6: *r* = 0.75; *P* < 0.0001 **(C)** LP-a: *r* = 0.71; *P* < 0.0001, **(D)** ox-LDL: *r* = 0.85; *P* < 0.0001, **(E)** TC: *r* = 0.64; *P* < 0.0001, **(F)** LDL: *r* = 0.80; *P* < 0.0001, **(G)** TG: *r* = 0.19; *P* = 0.146 (not significant), **(H)** HDL: *r* = −0.44; *P* < 0.001.

In pooled biochemical data, Lp-PLA_2_ correlated strongly and positively with TNF-α (*r* = 0.36; *P* < 0.01), IL-6 (*r* = 0.75; *P* < 0.0001), LP-a (*r* = 0.71; *P* < 0.0001), ox-LDL (*r* = 0.85; *P* < 0.0001), TC (*r* = 0.64; *P* < 0.0001), LDL (*r* = 0.80; *P* < 0.0001), TG (*r* = 0.19; *P* = 0.146), and inversely with HDL (*r* = −0.44; *P* < 0.00). These findings link Lp-PLA_2_ to both atherogenic lipoproteins and systemic inflammatory activation (Figure 2).

## Discussion

This study demonstrates that individuals with established atherosclerotic cardiovascular disease exhibit elevated circulating levels and whole-blood mRNA expression of Lp-PLA_2_, IL-6, and TNF-α, alongside increased oxidized lipoproteins and an atherogenic lipid profile. In addition, these findings are consistent with the well-established association between dysregulated lipid metabolism, oxidative stress, and systemic inflammation in cardiovascular disease.

Elevated IL-6 and TNF-α levels observed in this study are consistent with previous reports identifying these cytokines as key markers of vascular inflammation and cardiovascular risk (15, 16, 40). Both cytokines are known to be involved in inflammatory signaling pathways such as NF-κB and JAK/STAT, contributing to endothelial dysfunction and immune activation (12, 13, 41). However, in the present study, multivariable regression analyses demonstrated that these associations were largely explained by underlying metabolic factors, particularly LDL-C and glycemic status, rather than disease status alone. This suggests that circulating cytokine elevations may reflect the broader metabolic and inflammatory milieu rather than serving as independent indicators of atherosclerotic disease.

Likewise, the increased levels of Lp-PLA_2_ and its significant correlation with LDL-C, ox-LDL, and Lp(a) align with previous research emphasizing its function at the nexus of lipid oxidation and inflammation (5, 17, 20). Similarly, Lp-PLA_2_ breaks down oxidized phospholipids, which makes bioactive lipid mediators like lysophosphatidylcholine and oxidized fatty acids. These have been linked to signaling that causes inflammation. Nonetheless, the robust independent correlation between Lp-PLA_2_ and LDL-C identified in this study corroborates the notion that Lp-PLA_2_ predominantly signifies lipid-related inflammatory burden rather than serving as an independent causal mediator of atherosclerosis. This interpretation aligns with clinical and genetic studies indicating minimal causal effects of Lp-PLA_2_ inhibition on cardiovascular outcomes (29, 30, 42).

Importantly, a significant finding of this study is that, after controlling for BMI, LDL-C, HbA1c, and smoking, LDL-C and HbA1c were identified as the most reliable significant predictors after adjustment, although residual confounding cannot be excluded of Lp-PLA_2_ and inflammatory biomarkers, while the categorical CVD variable did not consistently persist. The results suggest that the biomarker discrepancies between groups are predominantly influenced by underlying metabolic dysfunction rather than solely by disease status. This underscores the necessity of accounting for cardiometabolic context when analyzing inflammatory and oxidative biomarkers in cardiovascular studies.

The simultaneous increase in protein levels and whole-blood mRNA expression of Lp-PLA_2_, IL-6, and TNF-α indicates a synchronized activation of inflammatory pathways. Nevertheless, caution is necessary when interpreting whole-blood mRNA data. Whole-blood gene expression indicates both transcriptional regulation and fluctuations in the composition of circulating leukocytes. However, in this study, CVD patients demonstrated neutrophilia and relative lymphopenia, which may affect the assessed expression levels. Therefore, the observed increases in mRNA expression cannot be attributed to cell-specific upregulation and do not directly reflect gene expression within the vascular wall.

Medication use is another important factor influencing interpretation. In addition, the CVD cohort exhibited a significant prevalence of statin and antidiabetic therapy, both of which are recognized for their ability to influence lipid and inflammatory biomarkers. Because medication use and disease status are closely linked, their independent effects could not be fully disentangled.

It's important to note a few limitations. The small sample size reduces its power and generalizability. The fact that only men were allowed to take part makes it less useful for women. Even though multivariable adjustment was done, it is still possible that there is residual confounding related to metabolic burden and medication use. Whole-blood mRNA expression also shows the composition of leukocytes, but it does not show gene expression in specific cells or blood vessels. Moreover, the comparison group was not entirely devoid of cardiometabolic risk factors, necessitating careful interpretation.

## Conclusion

In summary, individuals with diagnosed atherosclerotic cardiovascular disease demonstrate increased circulating and transcriptional indicators of inflammation and lipid oxidation, such as Lp-PLA_2_, IL-6, and TNF-α. Nonetheless, these associations are primarily elucidated by underlying metabolic factors, including dyslipidemia and glycemic status, rather than disease status alone. These results validate Lp-PLA_2_ as an indicator of lipid-related inflammatory activity and underscore the significance of metabolic context in the interpretation of cardiovascular biomarkers. Subsequent longitudinal studies with enhanced control of confounding variables and cell-specific analyses are necessary to elucidate mechanistic relationships. These findings should be interpreted with caution, as residual confounding cannot be excluded.

## Data Availability

The datasets presented in this study can be found in online repositories. The names of the repository/repositories and accession number(s) can be found in the article/Supplementary Material.
