# Supplementary material for: Elevated protein levels and mRNA upregulation of Lp-PLA2, IL-6, and TNF-α in cardiovascular disease among a Saudi population
Source: Front Cardiovasc Med. 2026 May 8;13:1774428. doi: 10.3389/fcvm.2026.1774428 (PMC13193930; doi:10.3389/fcvm.2026.1774428)
Supplement: Supplementary file 1 [file Table1.docx]

**Supplementary Table 1. Quantitative RT-PCR Primer Sequences**

| **Gene** | **Primer** | **Sequence (5′→3′)** |
| --- | --- | --- |
| **IL-6** | Forward | CAC CGG GAA CGA AAG AGA AG |
|  | Reverse | GGG CGG CTA CAT CTT TGG AAT C |
| **TNF-α** | Forward | AAG AAT TCA AAC TGG GGC CT |
|  | Reverse | GAG GAA GGC CTA AGG TCC AC |
| **Lp-PLA₂ (PLA2G7)** | Forward | CCA CCC AAA TTG CAT GTG C |
|  | Reverse | GCC AGT CAA AAG GAT AAA CCA CA |
| **GAPDH** | Forward | CAA GGT CAT CCA TGA CAA CTT TG |
|  | Reverse | GTC CAC CAC CCT GTT GCT GTA G |
